# CXCL13 in laboratory diagnosis of Lyme neuroborreliosis—the performance of the recomBead and ReaScan CXCL13 assays in human cerebrospinal fluid samples

**DOI:** 10.1007/s10096-021-04350-y

**Published:** 2021-10-09

**Authors:** Sofie Haglund, Malin Lager, Paula Gyllemark, Gärda Andersson, Oskar Ekelund, Martin Sundqvist, Anna J. Henningsson

**Affiliations:** 1 Department of Laboratory Medicine in Jönköping, Region Jönköping County, and Department of Biomedical and Clinical Sciences, Linköping University, Linköping, Sweden; 2 Department of Infectious Diseases, Region Jönköping County, and Department of Biomedical and Clinical Sciences, Linköping University, Linköping, Sweden; 3 Clinical Microbiology, Region Kronoberg, Sweden; 4 Department of Laboratory Medicine, Clinical Microbiology, Faculty of Medicine and Health, Örebro University, Örebro, Sweden; 5 Department of Clinical Microbiology in Linköping, Linköping University, Linköping, Sweden; 6 Department of Biomedical and Clinical Sciences, Division of Inflammation and Infection, Linköping University, Linköping, Sweden

**Keywords:** Lyme neuroborreliosis, Diagnostics, CXCL13, ReaScan, recomBead

## Abstract

The chemokine CXCL13 is used as complement to serology in the diagnostics of Lyme neuroborreliosis (LNB). We evaluated and compared the semi-quantitative, cassette-based ReaScan CXCL13 assay with the quantitative recomBead CXCL13 assay using a collection of 209 cerebrospinal fluid samples. The categorical agreement between results interpreted as negative, grey zone, and positive by the two methods was 87%. The diagnostic sensitivity was higher using the recomBead assay, whereas specificity was higher using ReaScan. Few manual steps, and a short turn-around time with no batching of samples makes the ReaScan CXCL13 assay an attractive complement to serology in the diagnostics of LNB.

**Supplementary Information:**

The online version contains supplementary material available at 10.1007/s10096-021-04350-y.

## Introduction

Lyme neuroborreliosis (LNB), the most common form of disseminated infection by bacteria in the *Borrelia burgdorferi* sensu lato complex in Europe [1], typically presents as cranial nerve palsy, sub-acute meningitis or other neurological impairments [2]. Definite LNB is defined as (i) neurological symptoms attributable to LNB, (ii) pleocytosis in cerebrospinal fluid (CSF, mononuclear cells > 5/µL CSF), and (iii) intrathecally produced anti-*Borrelia* antibodies (elevated antibody index, here referred to as positive Bb AI) [3].

The B cell-attracting chemokine CXCL13 has in previous studies been shown to be a useful and reliable complement to serology as it is elevated in the CSF of patients with very early LNB where the sensitivity of antibody tests may be low. Furthermore, CXCL13 has been proven useful in the monitoring of response to antibiotic treatment [4–7], and for the discriminating of acute LNB from other central nervous system (CNS) disorders [8–10]. However, the appropriate cut-off level varies between studies and has been debated [6–14].

The main aim of this work was to evaluate the rapid and semi-quantitative ReaScan CXCL13 assays (Reagena Ltd, Toivala, Finland) performance and compare it to the quantitative recomBead CXCL13 assay (Mikrogen Diagnostik, GmbH, Neuried, Germany), which was used in clinical routine in the laboratory in Jönköping as a complement to serology in selected cases and run once a week following batching of samples [15–17]. Secondly, we aimed to investigate the impact of the pre-analytical storage conditions on the test results.

## Materials and methods

### Study population

CSF samples (*n* = 209) from definite LNB (*n* = 53), probable early LNB (*n* = 16) and non-LNB (*n* = 140) patients were retrospectively included from the Clinical Microbiology Laboratories in Region Jönköping County, Region Kronoberg, and Örebro University Hospital. Patients with symptoms strongly suggestive of LNB, such as subacute meningitis or peripheral facial palsy, and CSF pleocytosis, but, due to short symptom duration, with negative Bb AI, were in our study classified as probable early LNB [3]. They were all considered as, and treated for, LNB by their physician and responded well to the treatment. If the criteria for definite or probable early LNB were not fulfilled, the patient was classified as non-LNB. The non-LNB group included patients both with and without CSF pleocytosis, whereof 22 had other confirmed CNS infections. Demographic and clinical data were retrieved from the patients’ medical records or the medical referral (Table 1). Samples were collected in tubes without additives and either analysed as fresh samples, or stored at − 20 °C before the CXCL13 analysis.

**Table 1:** Patient characteristics, *n* = 209

	CSF mononuclear cells > 5/µL CSF		CSF Bb AI		Age (years, mean, range)		Male (%)	Symptom duration (days, mean, range)
Definite LNB (53)	+	+		42 (2–87)		46		15 (1–280)^1^
Probable LNB (16)	+	−		21 (3–76)		47		3 (1–28)^2^
Non-LNB (140)	− (73)/ + (67)	−		47 (4–83)		49		30 (1–3650)^3^

*CSF* cerebrospinal fluid, *Bb AI* Borrelia specific antibody index, *LNB* Lyme neuroborreliosis. ^1^Data missing in three cases, ^2^data missing in three cases, ^3^data missing in 52 cases

### CXCL13 methods

The ReaScan CXCL13 is a rapid cassette-based immuno chromatographic system. The reader values are translated to semi-quantitative CXCL13 concentrations interpreted as < 250 pg/mL (negative), CXCL13 250–500 pg/mL (grey zone), and CXCL13 > 500 pg/mL (positive/suspected LNB), based on the instructions by the manufacturer.

In the quantitative recomBead CXCL13 assay, based on the Luminex xMAP® technology, samples are analysed in batches. The range of measurement is 9–1000 pg/mL. The CXCL13 concentration is interpreted as CXCL13 < 190 pg/mL (negative), CXCL13 190–300 pg/mL (grey zone), and CXCL13 > 300 pg/mL (positive/suspected LNB), based on the instructions by the manufacturer.

### Statistical analyses

Both the ReaScan and recomBead CXCL13 assays are CE/IVD validated by their manufacturers. Their recommended cut-off values were applied in the study, since these are most likely the cut-offs used by laboratories using these assays. Grey zone results were interpreted as positives in the evaluation of sensitivity and specificity, based on the tradition in our laboratory. In the calculations, patients with probable early LNB were considered as LNB, as they were diagnosed with and treated for LNB by their physician. The correlation between variables was investigated by Spearman rank correlation analysis. A *P* value < 0.05 was considered statistically significant. The impact of repeated freeze thawing (− 20 °C) and cold storage at 4–8 °C for up to one week was compared with results based on fresh samples with ReaScan CXCL13.

## Results

Overall, the categorical agreement between ReaScan CXCL13 and recomBead CXCL13 over the three CXCL13 concentration intervals was 87%. The reader values following analysis with ReaScan correlated with the CXCL13 concentration received after analysis with the recomBead assay; r_s_ = 0.76, *P* < 0.001.

The categorical agreement between the two methods is presented in Table 2. When all grey zone results were handled as positives, the overall agreement between the two methods was 90%; in negative samples it was 98%, and in positive samples 74%.

**Table 2:** Categorical agreement between the ReaScan and recomBead CXCL13 assays, with recomBead as reference (*n* = 209)

recomBead^1^			ReaScan^2^				Agreement (%)	
		Negative		Grey zone	Positive			
Negative	140	**137**		2	1			98
Grey zone	10	10		**0**	0			0
Positive	59	8		7	**44**			75

^1^recomBead CXCL13 negative; CXCL13 < 190 pg/mL, grey zone; CXCL13 191–300 pg/mL, positive; CXCL13 > 300 pg/mL. ^2^ReaScan CXCL13 negative; CXCL13 < 250 pg/mL, grey zone; CXCL13 250–500 pg/mL, positive; CXCL13 > 500 pg/mL

The diagnostic sensitivity, including the entire sample collection (*n* = 209), was lower for the ReaScan CXCL13 assay (78%) compared with the recomBead assay (86%). However, the specificity of ReaScan CXCL13 was higher (95% vs. 91%), (Table 3). In patients with probable early LNB, ReaScan showed a sensitivity of 43% and recomBead 50%.

**Table 3:** Results from all cerebrospinal fluid samples combined, analysed with the ReaScan CXCL13 assay and the recomBead CXCL13 assay, *n* = 209

	LNB (n = 69)^1^		Non-LNB (n = 140)^2^		Sensitivity		Specificity
ReaScan CXCL13							
Positive	54	7					
Negative	15	133		78%		95%	
recomBead CXCL13							
Positive	59	13					
Negative	10	127		86%		91%	

*LNB* Lyme neuroborreliosis. ^1^The LNB group comprised both definite LNB and patients with short symptom duration; probable early LNB. ^2^The non-LNB group included patients both with and without cerebrospinal fluid pleocytosis

In samples from patients with CNS infections other than LNB (*n* = 22), including 10 patients with tick-borne encephalitis (TBE), the categorical interpretation of the results differed between the methods in six cases (Supplementary Table 1).

The study of the impact of pre-analytical storage conditions on the ReaScan CXCL13 assays performance indicated that cold storage for up to three days or freeze thawing up to two cycles have minor effects on the analytical performance, at least at higher concentrations of CXCL13 (Supplementary Table 2).

## Discussion

In this study, which included CSF samples from patients classified based on clinical and laboratory parameters, the recomBead CXCL13 assay showed a somewhat higher diagnostic sensitivity, whereas the diagnostic specificity was slightly higher using ReaScan CXCL13. These observations are in agreement with previous comparisons between the ReaScan CXCL13 assay and enzyme-linked immunosorbent assays including non-LNB populations comprising a mix of patients with and without CFS pleocytosis [18, 19]. The specificity noticed in our comparison (95%) corresponded with the level suggested as sufficient for diagnostic accuracy by others [14].

The categorical agreement between the assays studied here was 87% overall, 98% in samples interpreted as negative using recomBead CXCL13, and 75% in positive samples. The observed discrepancies may be due to differences in antigen recognition by the detection antibodies used, a feature common to many unstandardised immunological assays aiming to detect the same antigen [20–22].

The correlation between the reader values of ReaScan CXCL13 and the concentration of CXCL13 measured using recomBead was strong (r_s_ = 0.76). However, samples over the measurement range of the recomBead CXCL13 assay were not further diluted in our comparison, and therefore the correlation may actually have been underestimated.

CXCL13 may increase in CSF in other inflammatory conditions of the CNS than LNB, however usually to lower concentrations [8–10]. When the performance of the assays was studied using samples from 22 patients with other CNS infections, including TBE, which is an important differential diagnosis to LNB, six discrepant results were noticed. Three samples, one from a patient with enterovirus meningitis and two with TBE, generated low values indicating negative results with ReaScan CXCL13, but grey zone or high concentrations with recomBead CXCL13. Another two samples, one from a patient with herpes simplex 2 meningitis and one with meningitis due to *Streptococcus pneumoniae*, showed grey zone results with the ReaScan CXCL13, but high concentrations with recomBead CXCL13. However, one sample from a patient with TBE generated a high value indicating a positive result with ReaScan, but a low concentration with recomBead. Taken together, these findings may reflect the higher specificity for LNB noticed for ReaScan CXCL13 in our study, but also that CXCL13 in TBE merits further investigation and that clinicians should be aware of the fact that CXCL13 may be elevated in the CSF from TBE patients as well.

The stability of the CXCL13 chemokine may be affected by the pre-analytical storage conditions, which may lead to erroneous results. Even though cold-storage for up to three days had minor impact on the results, our data indicate that samples should be stored frozen (− 20 °C) if it is not possible to analyse the fresh CSF samples immediately. This observation is supported by the results presented by Hytönen et al. [10]. They demonstrated that up to five freeze–thaw cycles did not affect the CXCL13 concentration significantly. However, those data were based on one sample only. Our samples did not undergo more than two freeze–thaw cycles, and therefore we can assume that they accurately reflect the CXCL13 concentration in fresh samples.

The time to an available test result using ReaScan CXCL13 was approximately 30 min, whereas the analytical turnaround time (TAT) for recomBead CXCL13 is approximately 4 h. Apart from the gain in TAT for the ReaScan CXCL13 assay, the possibility of immediate single-unit analysis of the samples after arrival to the laboratory, without batching of samples, which in our laboratory may be up to one week, is a major advantage for the ReaScan CXCL13 assay.

In conclusion, our results demonstrate that the recomBead and the ReaScan CXCL13 assays show comparable performance in the identification of LNB. The advantages with fewer manual steps and shorter TAT with no batching of samples makes the ReaScan CXCL13 assay an attractive alternative suitable even for smaller laboratories, and the rapid test results may support clinicians in their treatment decisions.

## Supplementary Information

Below is the link to the electronic supplementary material.

Supplementary file1 (DOCX 17.5 KB)

Supplementary file2 (DOCX 13.7 KB)

## Data Availability

Data and CSF samples may be available on reasonable request by contacting the corresponding author.
