# Supplementary material for: CXCL13 in laboratory diagnosis of Lyme neuroborreliosis—the performance of the recomBead and ReaScan CXCL13 assays in human cerebrospinal fluid samples
Source: Eur J Clin Microbiol Infect Dis. 2021 Oct 9;41(1):175–9. doi: 10.1007/s10096-021-04350-y (PMC8732935; doi:10.1007/s10096-021-04350-y)
Supplement: Supplementary file 1 — Supplementary file1 (DOCX 17.5 KB) [file 10096_2021_4350_MOESM1_ESM.docx]

**Supplementary Table 1** Results from the recomBead CXCL13 and ReaScan CXCL13 assays in patients with central nervous system infections of other etiology than *Borrelia burgdorferi* sensu lato

| Sample no | recomBead pg/mL | | recomBead interpretation^1^ | ReaScan reader-values | ReaScan pg/mL | | ReaScan interpretation^2^ | | Agreement between methods | CNS infection | | CSF cell count  leukocytes/µL | |  |
| --- | --- | --- | --- | --- | --- | --- | --- | --- | --- | --- | --- | --- | --- | --- |
| 1 | 67 | Neg | | 0 | | <250 | | Neg | Yes | | Enterovirus | | 14 | |
| 2 | <9 | Neg | | 0 | | <250 | | Neg | Yes | | Enterovirus | | 6 | |
| 3 | 53 | Neg | | 0 | | <250 | | Neg | Yes | | Enterovirus | | 260 | |
| 4 | 32 | Neg | | 0 | | <250 | | Neg | Yes | | Herpes simplex 2 | | 95 | |
| 5 | 253 | Grey zone | | 1 | | <250 | | Neg | No | | Enterovirus | | 265 | |
| 6 | 59 | Neg | | 1 | | <250 | | Neg | Yes | | Varicella zoster | | 12 | |
| 7 | 695 | Pos | | 166 | | >500 | | Pos | Yes | | Herpes simplex 1 | | 390 | |
| 8 | 119 | Neg | | 29 | | <250 | | Neg | Yes | | TBE | | 320 | |
| 9 | 119 | Neg | | 29 | | <250 | | Neg | Yes | | TBE | | 150 | |
| 10 | 60 | Neg | | 3 | | <250 | | Neg | Yes | | *Streptococcus pneumomiae* | | 10 848 | |
| 11 | 24 | Neg | | 4 | | <250 | | Neg | Yes | | Herpes simplex 1 | | 62 | |
| 12 | 464 | Pos | | 51 | | 250 - 500 | | Grey zone | No | | Herpes simplex 2 | | 669 | |
| 13 | 70 | Neg | | 6 | | <250 | | Neg | Yes | | TBE | | 80 | |
| 14 | >1000 | Pos | | 63 | | 250 - 500 | | Grey zone | No | | *Streptococcus pneumoniae* | | 2 846 | |
| 15 | 137 | Neg | | 7 | | <250 | | Neg | Yes | | TBE | | 276 | |
| 16 | 25 | Neg | | 0 | | <250 | | Neg | Yes | | TBE | | 32 | |
| 17 | 583 | Pos | | 0 | | <250 | | Neg | No | | TBE | | 276 | |
| 18 | 32 | Neg | | 1 | | <250 | | Neg | Yes | | TBE | | 32 | |
| 19 | 22 | Neg | | 0 | | <250 | | Neg | Yes | | Enterovirus | | 560 | |
| 20 | 257 | Grey zone | | 33 | | <250 | | Neg | No | | TBE | | 98 | |
| 21 | 56 | Neg | | 31 | | <250 | | Neg | Yes | | TBE | | 281 | |
| 22 | 28 | Neg | | 94 | | >500 | | Pos | No | | TBE | | 164 | |

CNS; central nervous system, CSF; cerebrospinal fluid, Neg; negative, Pos; positive, TBE; tick-borne encephalitis virus. ^1^recomBead CXCL13 Neg; CXCL13 < 190 pg/mL, grey zone; CXCL13 191 – 300 pg/mL, Pos; CXCL13 > 300 pg/mL. ^2^ReaScan CXCL13 Neg; CXCL13 < 250 pg/mL, grey zone; CXCL13 250 – 500 pg/mL, Pos; CXCL13 > 500 pg/mL
