# Supplementary material for: CXCL13 in laboratory diagnosis of Lyme neuroborreliosis—the performance of the recomBead and ReaScan CXCL13 assays in human cerebrospinal fluid samples
Source: Eur J Clin Microbiol Infect Dis. 2021 Oct 9;41(1):175–9. doi: 10.1007/s10096-021-04350-y (PMC8732935; doi:10.1007/s10096-021-04350-y)
Supplement: Supplementary file 2 — Supplementary file2 (DOCX 13.7 KB) [file 10096_2021_4350_MOESM2_ESM.docx]

**Supplementary Table 2** The impact of cold storage (4 – 8°C) and repeated freeze-thawing
(- 20°C) of cerebrospinal fluid samples on the interpretation of results (reader-values^1^) obtained from the ReaScan CXCL13 analysis

| Sample | Fresh | Class | 3 days 8°C | | Class *vs*. Fresh | 1 week 8°C | Class *vs*. Fresh | Freeze- thaw 1 cycle | Class *vs*. Fresh | Freeze- thaw 2 cycles | Class *vs*. Fresh |
| --- | --- | --- | --- | --- | --- | --- | --- | --- | --- | --- | --- |
| 1 | 94 | Pos | 117 | same | | 50 | Grey zone | 31 | Neg | 27 | Neg |
| 2 | 281 | Pos | 325 | same | | 312 | same | 252 | same | 270 | same |
| 3 | 380 | Pos | 443 | same | | 439 | same | 312 | same | 361 | same |
| 4 | 380 | Pos | 423 | same | | 367 | same | 95 | same | 252 | same |
| 5 | 438 | Pos | 449 | same | | 510 | same | 450 | same | 379 | same |
| 6 | 0 | Neg | 0 | same | | 0 | same | 0 | same | 0 | same |
| 7 | 0 | Neg | 0 | same | | 0 | same | 0 | same | 0 | same |
| 8 | 0 | Neg | 0 | same | | 0 | same | 0 | same | 0 | same |

^1^Reader-values < 40 corresponds to < 250 pg CXCL13/mL (Neg; negative result), and reader-values > 90 corresponds to > 500 pg CXCL13/mL (Pos; positive result)
